# Trimming tiers - Motivations and means for de-risking select agents

**DOI:** 10.3389/fbioe.2025.1630609

**Published:** 2025-06-27

**Authors:** Elizabeth Wells, Christopher Grisham, Michael T. Parker

**Affiliations:** ^1^ Department of Biology, Georgetown University, Washington, DC, United States; ^2^ College of Arts & Sciences, Georgetown University, Washington, DC, United States

**Keywords:** select agents, biosecurity, biological weapons, regulation, policy

## Abstract

In the United States, pathogens and toxins that pose a significant threat to public health are regulated via the Select Agents and Toxins list (SATL). Of those on the list, biological select agents and toxins (BSAT) deemed especially dangerous are designated “Tier 1”, and are subject to more stringent regulations. While general criteria for the addition of BSAT to the SATL have been published, along with criteria for Tier 1 designation, there are no clearly defined, publicly available steps for de-tiering or de-listing BSAT, making it difficult to decipher paths to risk reduction. We set out to better understand how the government has historically chosen to list and tier BSAT, to create a generalized profile of Tier 1 and non-Tier 1 BSAT, and to design a methodology that the government can utilize in efforts of de-risking BSAT. To these ends, we conducted a literature review compiling key information on all BSAT, with a specific focus on development and availability of vaccines and therapeutics, as well as evidence and/or reports of prior weaponization efforts. We then performed statistical analyses to compare Tier 1 BSAT to non-Tier 1 BSAT, allowing us to develop a “prototype” that describes the characteristics that are typical of each. Finally, we used these results to design a set of “priority” experiments and threshold goals for perceived risk criteria, the results of which enable clearer avenues to de-risking, and potentially also de-tiering and de-listing, of BSAT. Our results represent a call to action to bolster biosecurity through the utilization of BSAT prototyping, key experiments, and threshold implementation, all in an effort to enable evidence-based risk reduction of select agents.

## 1 Introduction

The select agent regulations (SARs) provide biosafety and biosecurity standards for the possession, use, and transfer of the most dangerous biological agents (Selectagents, 2025). An important aspect of the SARs is the select agents and toxins list (SATL), which identifies those biological select agents and toxins (BSAT) that pose a significant threat to the public health and safety of the United States, and for which there are strict regulatory control (Table 1). The SATL was established in 1996 by regulation from the Centers for Disease Control and Prevention (Department of Health and Human Services, 1996), resulting in a comprehensive list of 46 “select agents and toxins” as well as procedures for possession, transfer, disposal, and handling of these agents. The criteria for what constituted a BSAT was then described more concretely in the Public Health Security and Bioterrorism Preparedness and Response Act of 2002 (Congress, 2002), and generally considers the virulence or toxicity of an agent, its mode of transmission, and the availability of therapeutics. By 2008, the SATL had ballooned to include 79 agents (Animal and Plant Health Inspection Service, 2008; U.S. Department of Health and Human Services, 2008), and in 2010, Executive Order 13,546 set out to improve the program, in part by reducing the size of the list. The Departments of Health and Human Services (HHS) and United States Department of Agriculture (USDA) executed this task, ultimately reducing the overall size of the list and identifying (tiering) the most dangerous agents (President of the United States of America, 2010). The resulting SATL included 66 agents along with a “Tier 1” designation for the subset of agents that presented “the greatest risk of deliberate misuse with most significant potential for mass casualties or devastating effects to the economy, critical infrastructure, or public confidence” (President of the United States of America, 2010; Department of Agriculture, 2012; U.S. Department of Health and Human Services, 2012). Tier 1 agents and toxins are considered to be the most dangerous, and are subject to the most stringent regulations.

**TABLE 1:** Current select agents.

Non-tier 1 agents		Tier 1 agents	
Agent or toxin name	Type	Agent or toxin name	Type
Abrin	Toxin	Bacillus anthracis*	Bacterium
African swine fever virus^	Virus	*Bacillus cereus Biovar anthracis*	Bacterium
Avian influenza virus^	Virus	Botulinum neurotoxins	Toxin
Bacillus anthracis Pasteur strain*	Bacterium	Botulinum neurotoxin producing species of *Clostridium*	Bacterium
Chapare	Virus	Burkholderia mallei*	Bacterium
Classical swine fever virus^	Virus	Burkholderia pseudomallei*	Bacterium
Coniothyrium glycines (formerly Phoma glycinicola and Pyrenochaeta glycines)^	Fungus	*Ebolavirus*	Virus
Conotoxins (Short, paralytic alpha conotoxins containing the following amino acid sequence X1CCX2PACGX3X4X5X6CX7)	Toxin	Foot and Mouth Disease virus^	Virus
*Coxiella burnetii*	Bacterium	*Francisella tularensis*	Bacterium
Crimean-Congo haemorrhagic fever virus	Virus	Marburg Virus	Virus
Diacetoxyscirpenol	Toxin	Nipah virus	Virus
Eastern Equine Encephalitis virus	Virus	Rinderpest virus^	Virus
Far Eastern subtype	Virus	Variola major virus	Virus
Goat pox virus^	Virus	Variola minor virus	Virus
Guanarito	Virus	*Yersinia pestis*	Bacterium
Hendra virus	Virus	Adapted from https://www.selectagents.gov/sat/list.htm, accessed 03/14/2025	
Junin	Virus	* Overlap agents	
Kyasanur Forest disease virus	Virus	^ USDA agents	
Lassa fever virus	Virus		
Lujo virus	Virus		
Lumpy skin disease virus^	Virus		
Machupo	Virus		
Monkeypox virus	Virus		
*Mycoplasma capricolum^*	Bacterium		
*Mycoplasma mycoides^*	Bacterium		
Newcastle disease virus^	Virus		
Omsk hemorrhagic fever virus	Virus		
Peste des petits ruminants virus^	Virus		
*Ralstonia solanacearum^*	Bacterium		
*Rathayibacter toxicus^*	Bacterium		
Reconstructed replication competent forms of the 1918 pandemic influenza virus containing any portion of the coding regions of all eight gene segments (Reconstructed 1918 influenza virus)	Virus		
Ricin	Toxin		
*Rickettsia prowazekii*	Bacterium		
Rift Valley fever virus	Virus		
Sabia	Virus		
SARS-CoV/SARS-CoV-2 chimeric viruses resulting from any deliberate manipulation of SARS-CoV-2 to incorporate nucleic acids coding for SARS-CoV virulence factors	Virus		
Saxitoxin	Toxin		
*Sclerophthora rayssiae^*	Fungus		
Severe acute respiratory syndrome coronavirus (SARS-CoV)	Virus		
Sheep pox virus^	Virus		
Siberian subtype	Virus		
Staphylococcal enterotoxins (subtypes A,B,C,D,E)	Toxin		
Swine vesicular disease virus^	Virus		
*Synchytrium endobioticum^*	Fungus		
T-2 toxin	Toxin		
Tetrodotoxin	Toxin		
Venezuelan equine encephalitis virus	Virus		
*Xanthomonas oryzae^*	Bacterium		

The first iteration of Tier 1 presented a list of thirteen agents and toxins: eight agents were named by HHS, two by USDA, and three were shared overlap agents (Department of Agriculture, 2012; U.S. Department of Health and Human Services, 2012). Regulatory filings listed the twenty criteria by which each BSAT had been evaluated for Tier 1 consideration (Table 2) as well as the additional measures laboratories would need to follow to be approved to work with these agents and toxins (Table 3). The most impactful difference between working with general BSAT and Tier 1 agents is the need for heightened biosecurity measures. BSAT regulations require laboratories to designate a responsible individual, develop and implement a written security and biocontainment plans, and tune those plans to the perceived risk of the agent or toxin, given its intended use (7 CFR, 2025). For all of the above conditions (and more), Tier 1 agents carry more stringent requirements (Table 3). For example, thorough pre-access security assessments must be conducted for individuals to deem them suitable for Tier 1 access. Laboratories are instructed to restrict both the number of people who have access to a Tier 1 agent and the access to the laboratory outside of business hours. Entities that possess Tier 1 agents must also enforce extra security enhancements, including access limitations, extra physical barriers, intrusion detection systems, and visitation policies (Federal Select Agent Program, 2025). In addition, these laboratories are required to conduct annual insider threat awareness briefings on how to identify and report suspicious behaviors and to have a robust incident response plan that is much more rapid than that for other BSAT.

**TABLE 2:** Criteria to evaluate BSAT for Tier 1 status.

Criterion	
1	The relative ease with which a select agent or toxin might be acquired from a laboratory or commercial source
2	The relative ease of production of a select agent or toxin
3	The relative ease by which a select agent or toxin might be modified in order to enhance its pathogenicity, transmissibility, or ability to evade medical and non-medical countermeasures
4	The potential for easy deliberate dissemination
5	The potential for creating disease or illness
6	The relative environmental stability of a select agent or toxin by itself and how well it survives in the environment in which it is formulated or disseminated
7	The amount of select agent or toxin necessary to induce illness
8	The relative ease with which a particular select agent or toxin might be disseminated or transmitted from one animal or person to another or into the environment where it could produce a deleterious effect upon animal, plant, or human health
9	Whether the target population has innate immunity to the select agent or toxin or whether immunity has been acquired from a source such as vaccines
10	The potential for the select agent or toxin to create morbidity (i.e., any non-fatal illness that renders partial dysfunction to an animal or human lasting weeks or months that will eventually resolve with medical, veterinary, and/or supportive care)
11	The burden placed on the human, veterinary, or plant health system by the deliberate release of the select agent or toxin
12	The ability to detect a release of the select agent or toxin into the environment, food, water, or soil
13	The ability of the human and agricultural health authorities to accurately and rapidly diagnose and treat the disease presented by a release of the select agent or toxin
14	The existence of countermeasures to prevent, treat, or mitigate the symptoms of a disease caused by the release of a select agent or toxin and/or its spread through a population
15	The potential for high animal, plant, or human mortality rates with delivery of medical countermeasures
16	The potential for high animal, plant, or human mortality rates without delivery of medical countermeasures
17	The short-term economic impact of a single outbreak of a disease or release of a toxin
18	The human, monetary, and other resource costs of making an area, building, industrial plant, farm, or field safe for humans, animals or plants to inhabit following the release of the select agent or toxin
19	The pathogen’s ability to persist in the environment or to find a reservoir that makes its recurrence more likely
20	The long-term health or economic consequences caused by a single release of the select agent or toxin

Adapted from 77 FR, 61056.

**TABLE 3:** Regulations governing BSAT and additional regulations for Tier 1 agents.

BSAT	Additional tier 1 regulations
Security	
The security plan must be designed according to a site-specific risk assessment and must provide graded protection in accordance with the risk of the select agent or toxin, given its intended use	Establish a minimum of three security barriers, one of which must be monitored in such a way as to detect intentional and unintentional circumventing under all conditions
Criteria to be addressed include inventory control procedures, rules regarding cleaning, maintenance, and repair, provisions for securing select agents and tracking transfers or potential losses of select agents, and training employees in security procedures	Implement and test heightened incidence response plan that includes determining that the response time for security forces or local police will not exceed 15 min or that the established security barriers are sufficient to delay unauthorized access until the response force arrives
Eligibility	
All individuals working with select agents must undergo a security risk assessment and undergo proper training to work safely with said agent	Personnel must undergo ongoing suitability assessments which include self- and peer-reporting of any changes that may impact an individual’s ability to work with a Tier 1 agent
Access may be denied/revoked if the individual has been indicted/convicted of a felony; unlawfully uses controlled substances; is mentally defective or has been committed to a mental institution; has been dishonorably discharged from the US Armed Services; is illegally or unlawfully in the United States; or is associated through citizenship or membership with a country or organization designated as providing support for or actively participating in terrorism	Responsible for enhanced monitoring responsibilities, which includes holding annual insider threat awareness briefings on how to identify and report suspicious behaviors
	Required to provide additional training on entity policies and procedures for reporting, evaluation, and corrective actions concerning the assessment of personnel suitability
Biosafety	
Implement a written security plan and biocontainment plan that is commensurate with the risk of the select agent or toxin, given its intended use	Development of a biosafety plan that limits the number of individuals with access to Tier 1 BSAT based on each individual’s work objectives and limits the duration of time Tier 1 BSAT can be accessed
	The SRA approval process will serve as the first step for limiting access to select agents or toxins at the entity
Shipping and Transfer	
Proper documentation (APHIS/CDC Form 2) is required if transferring an identified select agent between two registered entities, one registered and one non-registered entity or importing into the US.	If Tier 1 select agents and toxins will be packaged in the shipping and receiving area, that area must meet all provisions associated with Tier 1 requirements. All individuals who will have access to Tier 1 select agents or toxins must also meet Tier 1 requirements and be enrolled in the entity’s suitability assessment program
Leadership	
Appoint a Responsible Official who is responsible for ensuring adherence to the regulations and inspecting facilities and procedures to ensure compliance	
Maintain detailed inventory of each agent sample, account for organisms exposed to/infected with select agents, record all individuals with access to areas containing agents, and document performed inactivation procedures	
Furnish a list of all select agents in use or intended for laboratory use, possession, or transfer, detailing the specific location within buildings the select agents will be stored	
The Responsible Official has increased demands to ensure safety and administrative compliances when working with a Tier 1 agent	

Adapted from 42 CFR § 73.11, 7 CFR § 331.11, and 9 CFR § 121.11.

While there exists a substantial description regarding the criteria by which agents are considered for Tier 1, there is a notable lack of criteria for de-tiering an agent, which may, in part, explain why the composition of Tier 1 has only been changed twice since its inception (added *Bacillus cereus* Biovar *anthracis* in 2017 and Nipah virus in 2024) (Department of Health and Human Services, 2017; Department of Health and Human Services, 2024). Similarly, there are no publicly disclosed criteria for de-listing a select agent. Given that BSAT and Tier 1 designations increase the difficulty with which researchers can study these agents and toxins, this regulatory one-way street presents a challenge for efforts to reduce the biosecurity risk posed by these high-consequence agents and toxins. The resultant heightened costs and concomitant decreased engagement in such research poses biosecurity risk as well, via reduced foundational knowledge and applied research integral to response efforts in the case of an outbreak or attack.

In this article, we strive to develop scientific and policy approaches for enabling the reduction of biosecurity risk posed by BSAT. First, we offer a brief review of the implications of SATL and Tier 1 status for practitioners, considering their dissuasion of research and contribution to reduced ability to generate knowledge of these agents and toxins. We then designed a “prototype pathogen” model for BSAT and Tier 1 agents, which enabled development of thresholds for criteria that, if met, could warrant reevaluation of BSAT status for individual agents entirely. Throughout this article, we will use the terms ‘de-tier,’ ‘de-list,’ and ‘de-risk’ to describe these approaches. De-tiering refers to the removal of an agent’s Tier 1 status, but the retention of its status as a select agent. De-listing refers to the removal of a pathogen from the SATL altogether. Finally, de-risking refers to reducing an agent’s potential to cause significant harm to the public, via increased knowledge of the agents, prevention and mitigation measures, etc. From these perspectives, we propose turning regulatory implementation on its head by using BSAT and Tier 1 status to prioritize certain kinds of research on these agents and toxins. The lessons learned are then adapted to current BSAT selection processes developed by the U.S. government, recommending criteria thresholds as goals for risk reduction. We conclude that these approaches would increase biosecurity through creating infrastructure for both de-risking BSAT overall and utilizing Tier 1 designation to prioritize scientific efforts to this end.

## 2 Motivations and strategies for de-risking BSAT

### 2.1 Agent selection, risk stasis, and biosecurity

Historically, the U.S. government has taken the lead on proposals for additions to and removals from the SATL. Since 2002, during each review of the SARs, HHS and USDA have considered the subject matter expertise of the Intragovernmental Select Agents and Toxins Technical Advisory Committee (ISATTAC), a group of experts spanning a broad swath of agencies of the U.S. government (CRS, 2025). In addition, notices of proposed rulemaking and proposed rules solicit the input of stakeholders via the public commenting process. These deliberations have historically been minimally forthcoming to the public eye, albeit recent publications detailing statistical decision frameworks are an important step in demystifying the process, if adopted long-term (Pillai et al., 2023; Pillai et al., 2022a; Pillai et al., 2022b).

It is important and difficult work to balance addressing the appropriate BSAT safety and security concerns with attempting to avoid overbearing restrictions that dissuade important scientific progress. This is made even more challenging by the fact that there is no mechanism for the evaluation of the efficacy of these policies in reducing biosecurity risk (Franz, 2015). So, while the SARs establish heightened biosafety and biosecurity standards for work with particularly dangerous agents and toxins, they may dissuade laboratories from undertaking work with BSAT because groups cannot justify incurring the additional burdens as compared to simply studying another organism (Gaudioso and Salerno, 1979; Casadevall and Relman, 2010; Dias et al., 2010; Wurtz et al., 2014; Centers for Disease Control and Prevention and United States Department of Agriculture, 2023; Centers for Disease Control and Prevention and United States Department of Agriculture, 2024; Kalra and Parker, 2022). On the government’s part these outcomes are at the very least expected, and at most a desired function.

Historically, there have been only vague criteria for how agents are considered for listing as BSAT, and no publicly disclosed decision-making process for removal or de-tiering of agents or toxins. This largely explains why BSAT may remain listed longer than justified by reasonable perceived risk analysis. Furthermore, much of the hard data on BSAT is either dated (often derived from now disbanded weapons research of the mid-20th century) or slowly evolving because of the stricture of regulation, so the addition and removal deliberations often rely strongly on inference of subject matter experts, which is subject to human biases and heuristics (Koblentz, 2011). Historically, the outcome is long-term residence of an agent or toxin on the SATL that in turn creates stagnant risk, where BSAT risk is bound within a range where the top end represents decreased overall risk (via less access and use) and the bottom end represents limitation on de-risking research. Such stagnation then positively feeds into a loop of continued residence on the SATL. Indeed, this is likely the case for some agents or toxins for which there have been previous weaponization efforts, but for which sanitation, medical countermeasures, etc. are today very effective (e.g., *Yersinia pestis*, Supplementary Table S1). It is notable that this issue correlates with the perceived risk of an agent or toxin and is thus particularly relevant for Tier 1 BSAT. Importantly, in the most recent biennial update (Department of Health and Human Services, 2024), improved clarity on criteria was provided, and this innovation is a welcome change that will lead to significant improvement in the process of listing/de-listing if maintained in the future.

The current situation that biases toward long-term residence of agents or toxins on the SATL is deleterious in multiple ways. Cost barriers to studying select agents may inhibit laboratories from engaging with de-risking research on these BSAT, and those laboratories that do conduct this research will be met with strict standards that decrease productivity. It follows that in the event of a BSAT public health crisis, it is likely that we will discover a reduced pool of expertise (Wurtz et al., 2014), resources, and preparedness for the BSAT, and thus a less effective public health response. We surmise that collectively, a lack of a methodology for de-tiering and de-listing BSAT is reducing public health security.

### 2.2 Parallels and lessons from other government tiering infrastructures

Two other government stratification scales are informative examples for this discussion. Similar to the SATL, the classifications of drugs by the Drug Enforcement Agency (DEA), called drug scheduling, stratifies substances into classes based on eight factors that collectively assess a drug’s potential for abuse as well as its medical value (United States Drug Enforcement Administration, 2025). This process involves posting proposed rules to the Federal Register to solicit public comments, followed by eventual final rules when it is determined that the drug will be scheduled in one of five categories. Importantly, once a drug is scheduled, it is difficult to change its schedule level, including de-scheduling. This is due to a variety of factors, including the variable application of the term “abuse”, international law, the need for large clinical trials to back up schedule decisions, etc. It is widely believed that the static nature of drug scheduling can be problematic, as it inhibits the ability to reassess drugs based on newly developed or discovered understanding of abuse likelihood and medical value (Lopez, 2016). However, changes to schedule (and de-scheduling) have both been accomplished through significant effort (Department of Justice, 2014; Department of Justice, 2022), primarily because of the clarity of the stated criteria for scheduling decisions.

The Fish and Wildlife Service (FWS) approach to the endangered species list is perhaps a more compelling example of adaptable list-based policy. Species are considered for listing as endangered (in danger of extinction in all or much of its natural range) or threatened (likely to soon become endangered) either by government proposal or stakeholder petition. After considering initial data, the FWS posts a proposed rule (or series of them) to the Federal Register to solicit additional biological data. If the data received in whole is compelling, then a final rule will be posted adding that species to the appropriate list (U.S. Fish and Wildlife Service, 2016). FWS describes their process to de-list species as an evaluation of achievements in eliminating or reducing threats, providing a clear two-way street (U.S. Fish and Wildlife Service, 2011). These clear criteria for de-listing (which are the same as those considered for initial listing) paired with consideration of public feedback ultimately guides the FWS’ de-listing decisions. On the whole, it is evident that the combination of clear criteria and de-listing processes bring efficiency to initial evaluation as well as reevaluation, and that the listing of a species is a call to action for the government and public to take steps to protect organism(s) and to participate in risk reduction.

### 2.3 A flipped approach: strategies for de-risking BSAT

Collectively, the adaptability of the SATL is slightly greater than that of drug scheduling but markedly less than that of the endangered species list. Sparse SATL criteria make for uneven and confusing implementation; government control enables listing and de-listing but in practice effectuates more of the former than the latter. The public’s feedback is taken into consideration via the Federal Register’s public commenting process but seems to be given less weight than opinions of government insiders, and inclusion on the SATL inhibits research rather than providing initiative for taking steps toward de-risking. While the SATL is intended to promote biosafety and biosecurity by limiting the scope and breadth of experiments on the most dangerous agents and toxins, the effect is instead somewhat paradoxical. By dampening feasibility and motivation to study BSAT, the SARs fix BSAT risk at heightened levels. This begs the question: If the most dangerous agents and toxins are defined, would it be useful to *prioritize* efforts key to reducing their overall threat, rather than inhibiting them?

To address this point, we contend that there is untapped potential in the SARs; it would be useful to flip the approach to use the BSAT and Tier 1 designations as a mechanism to prioritize specific types of research to better understand biological properties in support of risk assessments and decisions. This type of approach already exists in the U.S. government via the Probabilistic Analysis for National Threats Hazards and Risks (PANTHR) program (Department of Homeland Security, 2025), which endeavors to fill knowledge gaps on threat agent characteristics and the effects of technological advance. To better enable synergy between the SARs, PANTHR, and other gap analysis methods in the government, we set out to develop approaches with an ethos of action and risk reduction. In the remainder of this manuscript, we present three strategies to accomplish this aim: prototype BSAT modeling, priority experiments for de-risking, and threshold (goal) development for de-tiering, de-listing, and de-risking.

## 3 Prototype BSAT modeling

### 3.1 Pathogen prototyping as a means for de-risking BSAT

The National Institute of Allergy and Infectious Diseases (NIAID) has proposed the implementation of a “prototype pathogen model” for pandemic preparedness (Cassetti et al., 2023). The foundational idea for the model is that current methods of researching individual pathogens can be used as models for work on related pathogens. As such, NIAID identified one or multiple “prototype pathogens” for known human-infecting virus families believed to have pandemic-causing potential. These surrogates are then proposed as models for which experimental results can be applied to their respective families. Each family was also identified as having certain key gaps of information that need to be filled, and as such would require research and experimentation with the goal of addressing those gaps. Overall, using these inferences could prove useful in increasing pandemic preparedness for key groups of viruses that pose the greatest perceived risk for causing outbreaks.

Here, we adopt a prototyping model for BSAT with similar goals, but a different approach. Similar to NIAID, we sought to identify experimental data gaps that can be addressed, allowing development of research agendas to meet those deficiencies. However, our approach differed in that it aimed to categorize BSAT across microbial classes to better understand the “why?” of listing and stratification of select agents, from a security analysis perspective. In doing so, we identified themes for agent selection, allowing us to probe the merits of those themes as well as to develop approaches to direct decision-making qualitatively, based on observing changes in biosecurity risk over time. These approaches thus provide an action-oriented mindset, allowing for research prioritization to de-risk these agents.

### 3.2 Methods

#### 3.2.1 Criteria

We first sought to understand how BSAT have historically been tiered. To this end, we examined the twenty criteria utilized for the tiering of agents and toxins in 2012 (Table 2; Department of Agriculture, 2012; U.S. Department of Health and Human Services, 2012), and from that list developed a short list of criteria that, in our estimation, were most likely to impact Federal Select Agents Program (FSAP) decisions to tier agents and toxins in the past, present, and future. These criteria are:

• Vaccine development
• Vaccine availability
• Therapeutic development
• Therapeutic availability
• Therapeutic efficacy
• Interhost transmission
• Untreated mortality rate

The existence and availability of a safe and effective vaccine stands as one of the most important factors that can determine the perceived risk profile of an agent, and considerations in vaccine status should exist as a foundational criterion for de-risking. Similarly, analysis of the sufficiency of therapeutic development, efficacy, and availability provides insight into the threat posed by a BSAT event. By extension, we expected that the perceived threat posed by a BSAT would be strongly correlated with both interhost transmissibility (due to the ability this endows to produce larger outbreaks of disease) and the untreated mortality rate (due to both the biological and psychological effects associated with high mortality agents).

#### 3.2.2 Additional factors

While these criteria directly relate to those outlined by the government, a more robust prototype model necessitates incorporating additional factors as well, such as agent type (bacteria, fungus, toxin, virus), host tropism, and prior weaponization efforts, the last of which is a component of some of the criteria, but has been documented by various policy groups to be a generally “unstated” consideration of select agent deliberations (National Academies, 2010; The Scientist, 2022; Williams et al., 2025).

While both BSAT type and host tropism are not directly correlated with perceived threat, we hypothesized that these factors do exert strong influence on the SATL, as reflected in the proportions of various agent and toxin types on the list and the proportion of BSAT that infect humans compared to other hosts. Additionally, we expected that previous efforts to weaponize an agent or toxin would strongly correlate with both BSAT and Tier 1 designations.

#### 3.2.3 Analysis

We conducted a literature review examining properties of different subgroups of the SATL: Tier 1 BSAT, non-Tier 1 BSAT, human-tropic Tier 1 BSAT, and human-tropic non-Tier 1 BSAT (also considering human-specific and overlap BSAT separately). Each agent was individually researched and evaluated on the criteria and additional factors listed above and then analyzed mathematically to perform inter-group comparisons.

For criteria/additional factors where agents were sorted into binary categories (e.g., whether an agent was weaponized previously or not), we used an odds ratio (OR) analysis to determine differences between the subgroups of the SATL. Generally, the OR describes an association between risk factors and outcomes, and specifically in this study is used to evaluate whether agent groups differ significantly in a certain risk criteria or additional factor. We used Tier 1 as the reference group, meaning that the OR calculations represent the odds of a criteria or additional factor to be attributed to a Tier 1 agent compared to a non-Tier 1 agent. An OR = 1 implies that Tier 1 and non-Tier 1 agents are equally likely to contain a certain characteristic. When OR > 1, this suggests that Tier 1 agents are more likely to have a certain characteristic than non-Tier 1 agents. Conversely, an OR < 1 implies that Tier 1 agents are less likely to have a certain characteristic than non-Tier 1 agents. Precedent in clinical epidemiology studies suggest that an OR ≥ 3 (or equivalently, ≤0.33) is considered a delineating characteristic between groups, and so we defined prototypical characteristics of either Tier 1 or non-Tier 1 agents as those with [OR ≤ 0.33] or [OR ≥ 3] (Tugwell et al., 2012).

There were two categories with non-binary data distributions: untreated mortality and agent type. Untreated mortality was represented as percentages, and as such we conducted an unpaired T-Test. This statistical analysis shows whether there is a significant difference in the means of two groups of equal variances (mean untreated mortality in both Tier 1 and non-Tier 1 agents) (Albright, 2021). A relevant caveat to this statistical analysis is that Tier 1 consists of only 15 agents, limiting the power of the results of statistical analysis. Finally, to represent prototypical characteristics based on BSAT type (bacteria, fungus, toxin, virus), we used raw percentages, as the data is not appropriate for OR analysis or a T-test. Note that *Bacillus cereus* biovar *anthracis* and SARS-CoV-1/SARS-CoV-2 chimeric viruses were excluded from therapeutic efficacy and untreated mortality rate analyses, as neither agent has a reported case of infection in humans (The American Society for Microbiology, 2017; Wells and Parker, 2023).

### 3.3 Modeling results

The overall results of our literature review are listed in Supplementary Table S1 and Table 4, with Supplementary Table S1 providing the sourced data and Table 4 showing the percentages of Tier 1 and non-Tier 1 agents that fit the aforementioned criteria and additional factors. Further, Table 5 uses odds ratio (OR) calculations to show whether differences between Tier 1 and non-Tier 1 agents meet our threshold for prototype characteristics. The results of each criterion and additional factor will be discussed in individual sections.

**TABLE 4:** Rates of criteria and additional factors for prototype BSAT.

Categories			Vaccine		Therapeutic			Interhost transmission (%)	Untreated mortality (%)
			Developed (%)	Available (%)	Developed (%)	Available (%)	Effective (%)		
Criteria	Tier 1	Human only	69.2	30.8	84.6	84.6	66.7	46.2	48.2
		Human/Animal	73.3	40.0	73.3	73.3	57.1	53.3	48.4
		Overall	73.3	40.0	73.3	73.3	57.1	53.3	48.4
	non-Tier 1	Human only	25.8	16.1	12.9	12.9	10.0	32.3	16.0
		Human/Animal	42.9	16.7	14.3	14.3	7.3	50.0	23.3
		Overall	37.5	14.6	12.5	12.5	6.4	56.2	24.0

Categories			BSAT Type				Human-Affecting (%)	Prior Weaponization Efforts (%)
			Bacteria (%)	Fungi (%)	Toxin (%)	Virus (%)		
Additional Factors	Tier 1	Human only	53.8	0.0	7.7	38.5	100.0	76.9	
		Human/Animal	46.7	0.0	6.6	46.7	86.7	80.0	
		Overall	46.7	0.0	6.6	46.7	86.7	80.0	
	non-Tier 1	Human only	9.7	0.0	25.8	64.5	100.0	54.8	
		Human/Animal	11.9	0.0	19.0	69.1	73.8	54.8	
		Overall	16.7	6.3	16.7	60.3	64.6	52.1	

**TABLE 5:** Odds ratio of criteria and additional factors for prototype BSAT.

Criteria	All agents	Plants excluded	Human infecting only
Vaccine Development	4.58^a^	3.67^a^	6.47^a^
Vaccine Availability	3.90^a^	3.33^a^	2.31
Therapeutic Development	19.25^a^	16.50^a^	37.13^a^
Therapeutic Availability	19.25^a^	16.50^a^	37.13^a^
Therapeutic Efficacy	19.56^a^	16.89^a^	18.00^a^
Interhost Transmission	0.89	1.14	1.80
Untreated Mortality Rate	^b^		

Additional Factors	All Agents	Plants Excluded	Human Infecting Only
Host Tropism	^c^		
Infects Humans	3.56^a^	2.31	-
Prior Weaponization Efforts	3.68^a^	3.30^a^	2.75

^a^ [OR ≤ 0.33] or [OR ≥ 3].

^b^ T-Test used in place of OR.

^c^ Percentages used in place of OR.

#### 3.3.1 Vaccine development and availability

For each BSAT, we reviewed the literature to ascertain current vaccine development and vaccine availability. Vaccine development refers to whether or not a vaccine has surpassed a Phase 2 clinical trial in the U.S. or alternatively received full licensure in foreign countries. Vaccine availability considers if large quantities are available (or can rapidly be made so via international or domestic sourcing) to the general host population in the U.S. (Supplementary Table S1).

**FIGURE 1:**
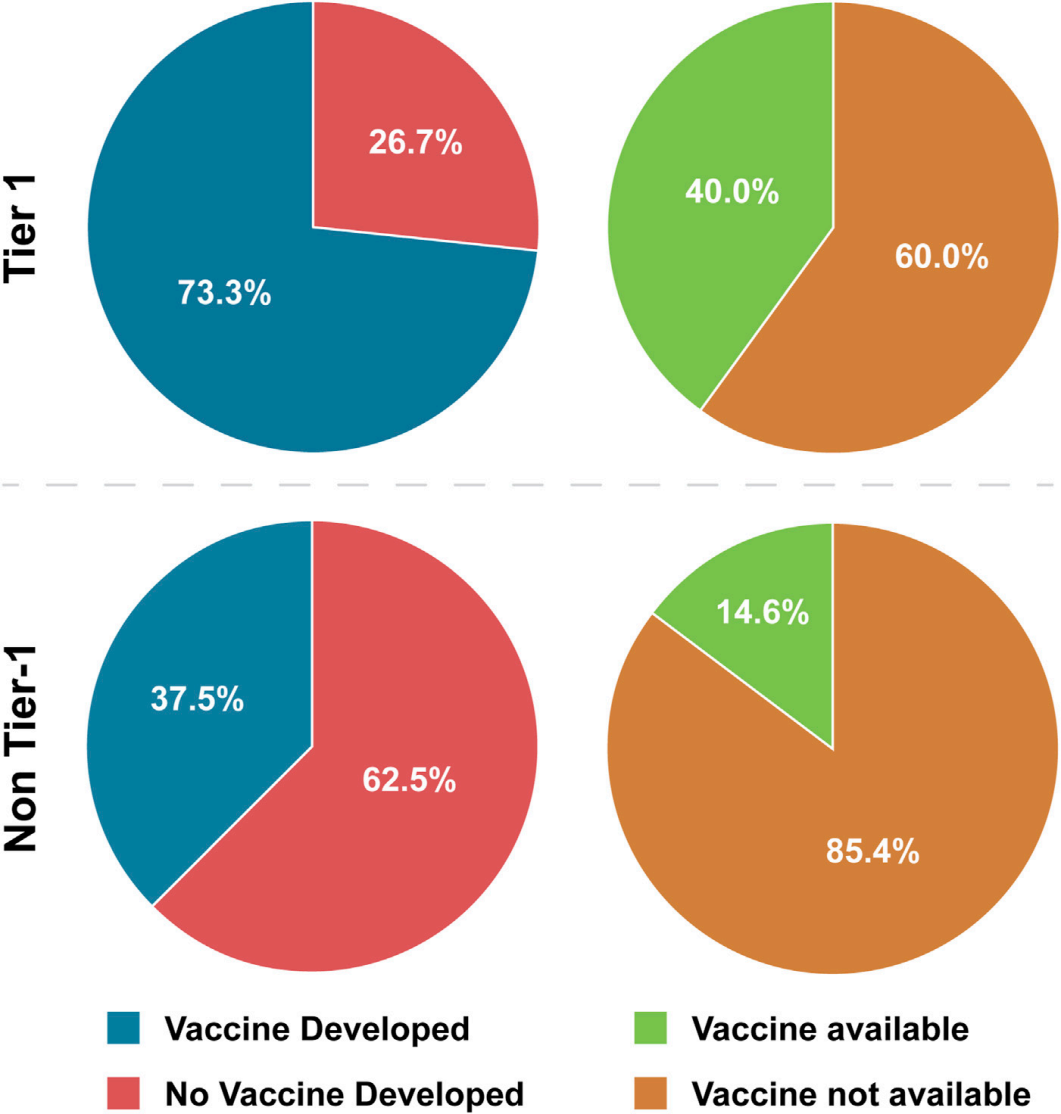
Tier 1 versus Non-Tier 1 Vaccine Development and Availability. Comparison of the number of BSAT across categories of vaccine development and availability.

This analysis showed that vaccines have been developed for 73.3% of Tier 1 BSAT and 37.5% of non-Tier 1 BSAT (Table 4; Figure 1). Considering that no plant-affecting BSAT are Tier 1, we conducted further analysis removing this group in order to determine if it was skewing the results. This additional analysis found that vaccines have been developed for 42.9% of non-Tier 1 human- and animal-affecting BSAT (an increase of 5.4% compared to the baseline; Table 4). However, when we calculated the odds ratio for development in Tier 1 versus non-Tier 1 agents, the exclusion of plants made no difference, as both calculations met our thresholds [OR ≤ 0.33] or [OR ≥ 3] for a strong association (Table 5). The results for vaccine availability also met the OR thresholds for a strong association with Tier 1 BSAT (40%) versus non-Tier 1 BSAT (14.6%) by OR calculation (Table 4; Figure 1), but are not sufficient for prototypical labeling because they did not represent the majority of their groups. We thus conclude that vaccine development is typical of Tier 1 BSAT, differentiating it from non-Tier 1, while vaccine availability is typical for neither Tier 1 nor non-Tier 1 BSAT (Figure 6).

#### 3.3.2 Therapeutic development, availability, and efficacy

To analyze the status of therapeutics for BSAT, we conducted a literature review to find information on therapeutic development, availability, and efficacy (Supplementary Table S1). These subcategories, respectively, evaluate whether a therapeutic:

1. Has been developed and tested for safety in the target host
2. Is available or can be quickly made available at scale to the public
3. Can lower mortality of an agent by at least 5%

**FIGURE 2:**
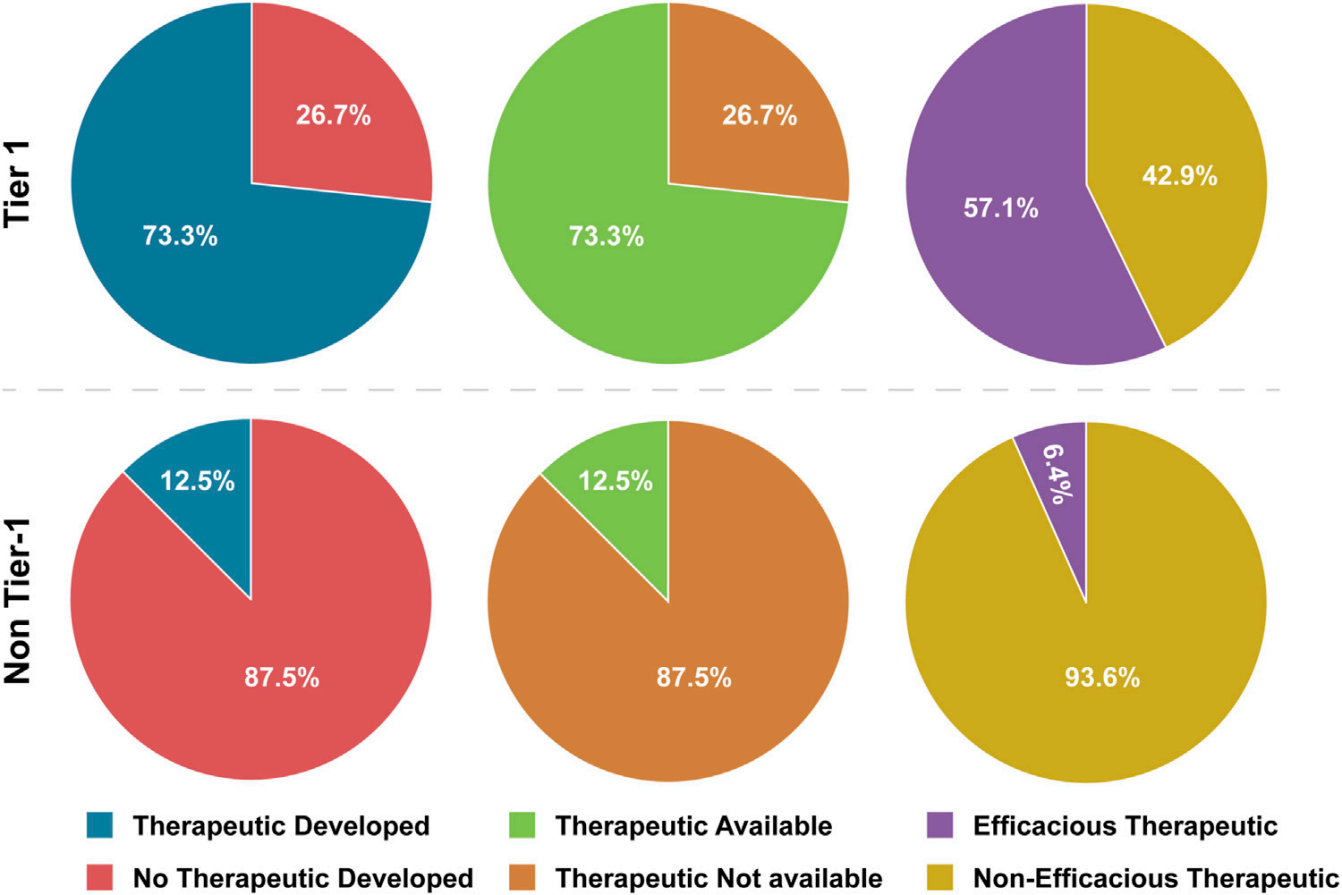
Tier 1 versus Non-Tier 1 Therapeutic Development, Availability, and Efficacy. Comparison of the number of BSAT across categories of therapeutic development, availability and efficacy. Note that *Bacillus anthracis* therapeutics are assumed to work for *Bacillus cereus* Biovar *anthracis* (Tier 1) infections, so this agent is included in development and availability calculations, but not in efficacy calculations (because no infections in humans have been documented). SARS-CoV-1/SARS-CoV-2 chimeric viruses (non-Tier-1), which have not been documented to exist, are excluded from all therapeutic calculations.

Therapeutics have been developed for 73.3% for Tier 1 BSAT and only 12.5% of non-Tier 1 BSAT (Table 4; Figure 2). When we examined therapeutic development in human-affecting BSAT only, the results were similar; therapeutics have been developed for 84.6% of human affecting Tier 1 BSAT and 12.9% of human affecting non-Tier 1 BSAT. Statistical analysis showed that the existence of a developed therapeutic is 19.25 times more likely for Tier 1 agents than non-Tier 1 agents. This OR exceeds our threshold and continued to do so both when plants were excluded and when only human-affecting agents were analyzed (Table 5).

We observed large differences between Tier 1 and non-Tier 1 therapeutic availability and efficacy as well. Therapeutics are available for 73.3% of Tier 1 BSAT versus 12.5% of non-Tier 1 BSAT (Table 4; Figure 2). OR analysis demonstrated that Tier 1 agents are 19.25 times more likely than non-Tier 1 agents to have an available therapeutic. The OR remained above 3 both when plants were excluded and when only human-affecting agents were analyzed (Table 5). Additionally, effective therapeutics (those that reduce mortality by at least 5%) are available for 57.1% of Tier 1 BSAT versus 6.4% of non-Tier 1 BSAT. Tier 1 agents are 19.56 times more likely to have an effective therapeutic than non-Tier 1 agents, and again this analysis met our threshold both when plants were excluded and when the analysis was limited to just human-affecting agents. Collectively, our analysis demonstrates that a developed therapeutic that is both available and efficacious against the agent is typical of Tier 1 BSAT, and these are differentiating characteristics in comparing them to non-Tier 1 BSAT (Figure 6).

#### 3.3.3 Interhost transmission

This criterion refers to the passing of a pathogen from one host to another. While the SATL encompasses a diverse set of mechanisms by which pathogens are spread among hosts, ongoing chains of transmission outside of the initial inoculation are a particularly important criterion that increases an agent’s risk. “No transmission” refers to agents that cannot be passed directly from host to host (R_0_ = 0), typical of dead-end infections and toxins. While some select agents have been assumed to be contagious, if evidence does not exist to support these assertions, we placed that agent into this category. The “Transmission” category thus captures all agents that have an R_0_ > 0, and within this group we evaluated two subcategories. Non-sustained transmission refers to agents that can be passed from host to host, but each infected host infects on average less than one other host (0 < R_0_ < 1). This is more typical of infections that rely on direct blood contact, aerosolized excrement or urine, etc. Conversely, sustained transmission refers to agents that can create chains of spread from host to host; more specifically, each infected host infects on average one or more other host (R_0_ > 1). While considering these subcategories separately at first, we concluded that from a prototyping perspective, it was more logical to combine the two for our analysis, because of the seeming importance within the SATL toward the characteristic of any interhost transmission.

**FIGURE 3:**
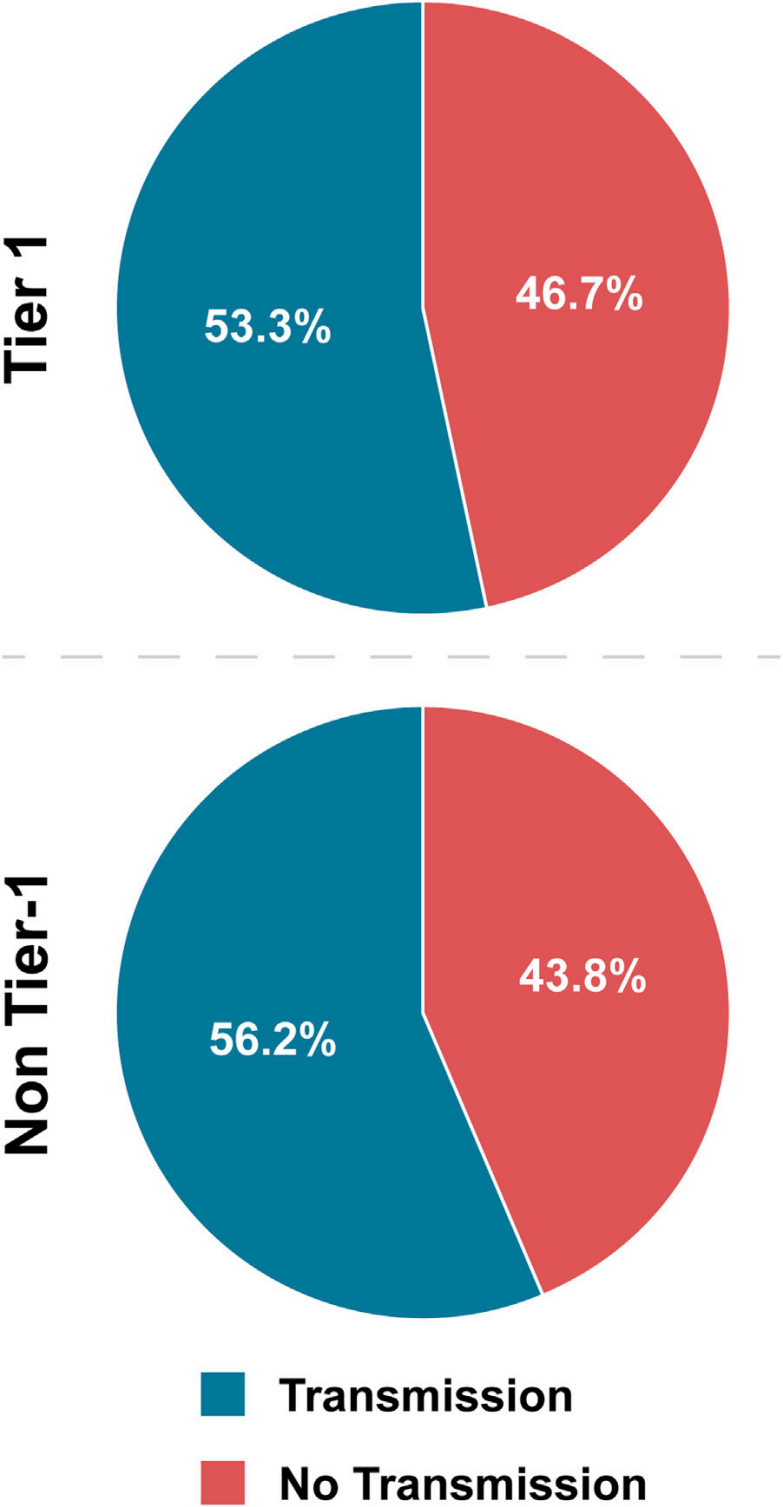
Tier 1 versus non-Tier 1 Interhost Transmission. Comparison of the number of BSAT that exhibited the characteristic of interhost transmission in each group.

Overall, 53.3% of Tier 1 agents have interhost transmission (combined sustained and non-sustained), whereas 56.2% of non-Tier 1 agents fall into the same category (Table 4; Figure 3). These values are not significantly different by OR evaluation, and because the majority of agents in both categories exhibited this characteristic, we conclude that interhost transmission is a prototypical characteristic of both Tier 1 and non-Tier 1 BSAT (Figure 6).

#### 3.3.4 Untreated mortality rate

While data on untreated mortality rate is presented similarly for human- and animal-affecting BSAT, plant mortality is more commonly expressed as percent (%) yield loss. For the purposes of our analysis, we thus use % yield loss as an estimation of plant “mortality.” It is worth noting that *Bacillus cereus* Biovar *anthracis* (Tier 1) and SARS-CoV-1/SARS-CoV-2 (non-Tier 1) were excluded from this calculation, as neither have had a reported infection in humans, making calculation of untreated mortality rate impossible.

The mean untreated mortality rate for Tier 1 BSAT is 48.4% whereas the untreated mortality rate for non-Tier 1 BSAT is 24.0% (Table 4). A two-tailed T-test conducted using all agents produced a p < 0.05 (p = 0.0055; dof = 58), which indicates that the mean untreated mortality rate of Tier 1 and non-Tier 1 agents differ significantly. The same statistical analysis was performed while excluding all non-human infecting agents (p = 0.000231; dof = 39) which also produced p < 0.0005. From this, we determined that the prototypical Tier 1 BSAT has an untreated mortality rate above 40%, whereas the prototypical non-Tier 1 BSAT has an untreated mortality rate below 40% (Figure 6).

#### 3.3.5 BSAT type

**FIGURE 4:**
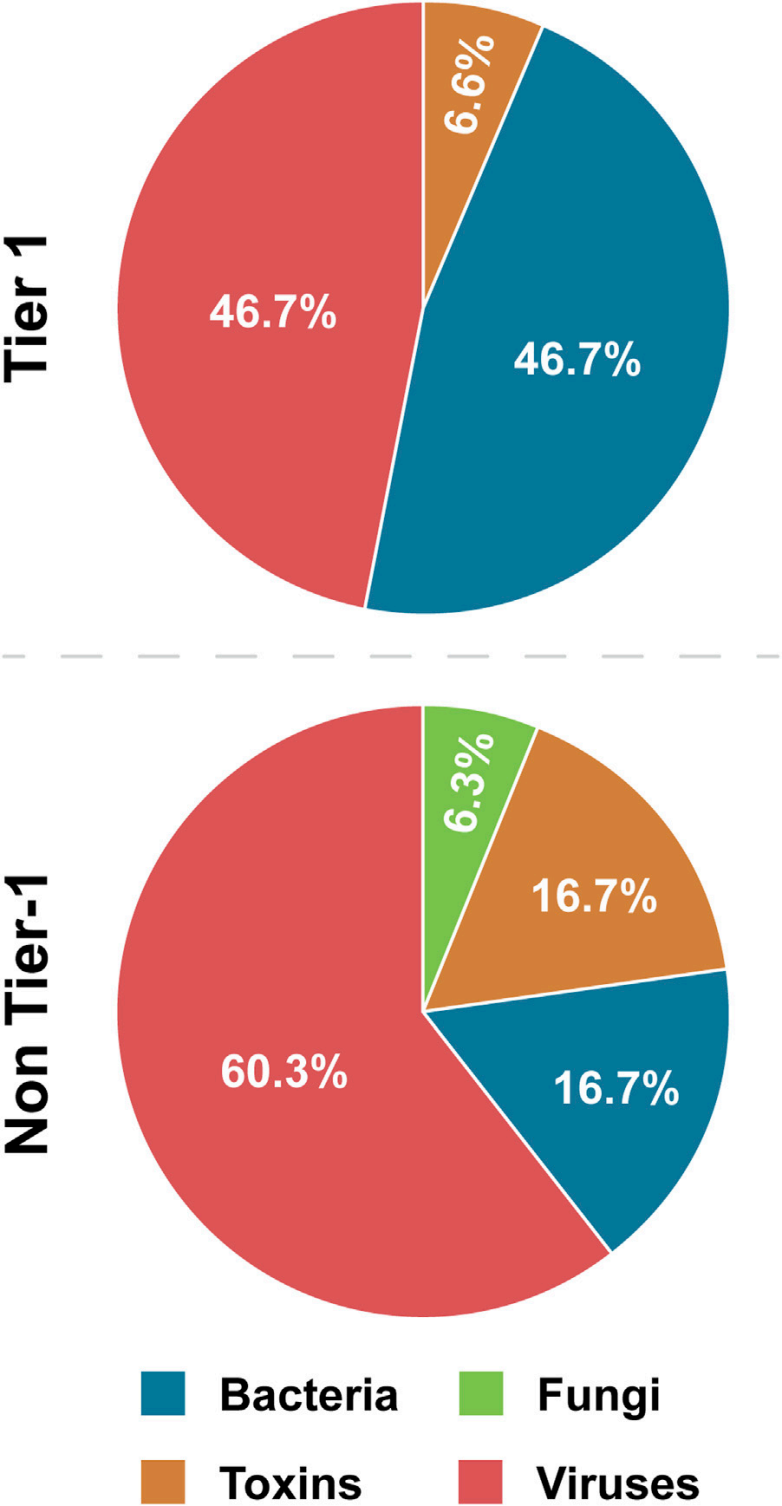
Tier 1 versus non-Tier 1 Agent Type. Comparison of the number of BSAT across four categories of agent type: bacteria, fungi, toxins, and viruses.

This factor refers to whether a BSAT is a bacterium, fungus, toxin, or virus. Bacteria and viruses are the most common BSAT, together constituting 81.0% of the SATL, and 93.4% of Tier 1 (Table 4; Figure 4). Bacteria are overrepresented in Tier 1 (46.7%) compared to non-Tier 1 (16.7%), while viruses are overrepresented in non-Tier 1 (60.3%) compared to Tier 1 (46.7%). Thus, the prototypical Tier 1 BSAT is either a virus or bacterium, and the prototypical non-Tier 1 BSAT is a virus (Figure 6).

#### 3.3.6 Host tropism

We evaluated this factor by identifying the primary host(s) for which a BSAT was listed, either humans, animals, humans and animals, or plants. While 86.7% of Tier 1 BSAT are human-affecting, that value falls to 64.6% for non-Tier 1 BSAT. Further, no Tier 1 BSAT are plant pathogens, and 40.0% of Tier 1 BSAT affect animals (Table 4). When all agents are included, Tier 1 agents are 3.56 times more likely to infect humans than non-Tier 1 agents, and when plants are excluded, Tier 1 agents are 2.31 times more likely to infect humans than the remaining non-Tier 1 agents. Only the former of these results meets our threshold for a differentiating characteristic (Table 5). Overall, we conclude that both the prototypical Tier 1 BSAT and non-Tier 1 BSAT are human-affecting (Figure 6).

#### 3.3.7 Prior weaponization efforts

While the twenty criteria outlined by FSAP for the tiering of BSAT do not overtly reference prior weaponization of an agent, criteria #3 and #4 (Table 2) clearly consider this topic. Further, we and others have reported that prior weaponization efforts; inclusive of attempts to acquire, attempts to develop, and success in development; are clearly factors that are considered by FSAP when determining inclusion on the SATL, as well as tiering of BSAT (National Academies, 2010; The Scientist, 2022). Prior weaponization efforts, beyond proof of technological viability, can create precedent that potentially elicits interest from future weaponeers, and increases concern of redevelopment on the part of security practitioners and the public. For example, the anthrax attacks not only demonstrated the feasibility of weaponization of anthrax, but created a sense of fear in the American public of subsequent attacks (CDC, 2025).

**FIGURE 5:**
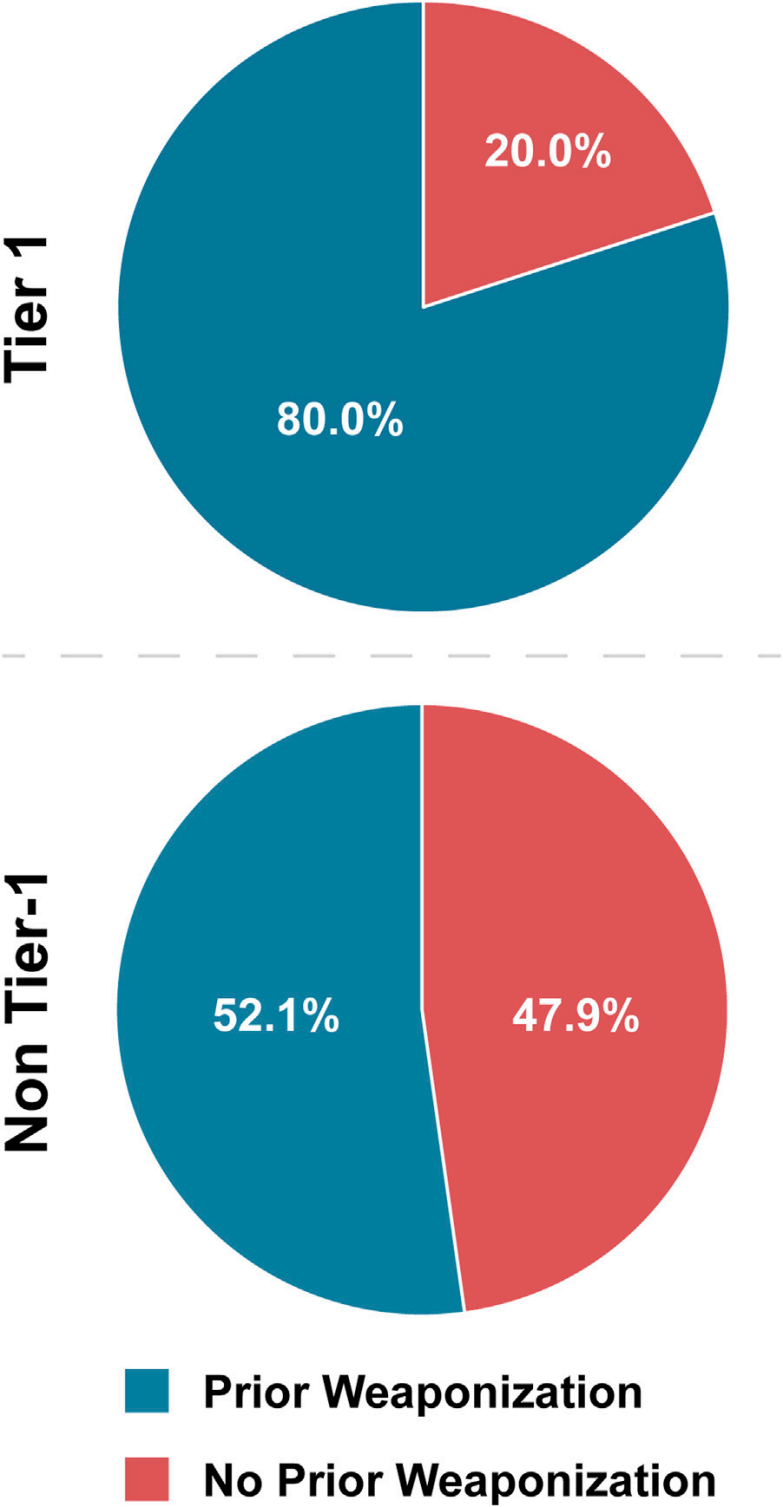
Tier 1 versus non-Tier Prior Weaponization Efforts. Comparison of the number of BSAT for which evidence exists publicly that weaponization has been pursued, inclusive of attempts to acquire, attempts to develop, and success in development.

**FIGURE 6:**
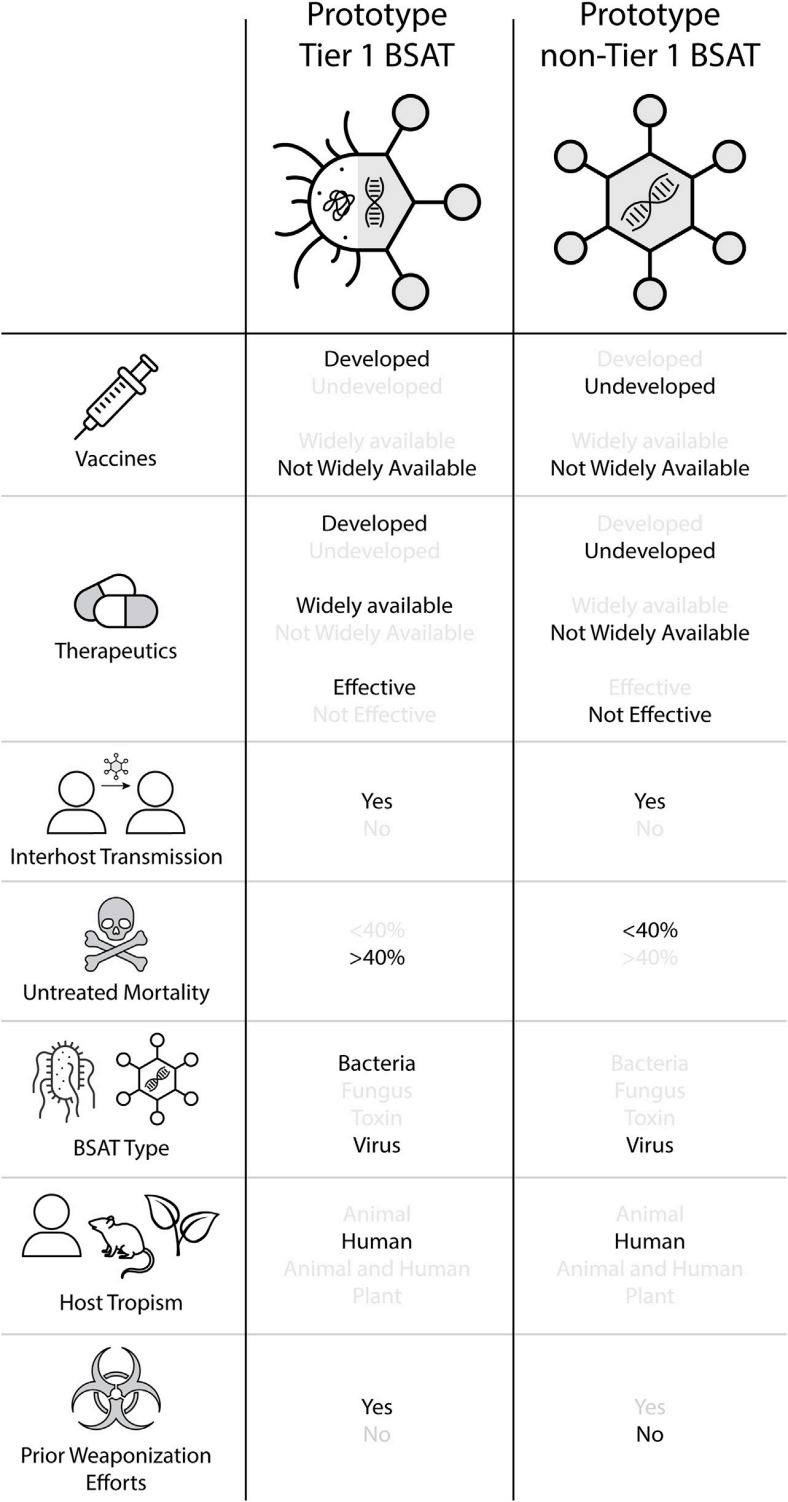
The Prototypical Tier 1 and non-Tier 1 BSAT. Results are indicated in black lettering, while counterfactuals are gray. Notably, these prototypes are dissimilar in the following categories: vaccine development; therapeutic development, availability, and efficacy; untreated mortality; prior weaponization efforts, and (to some extent) BSAT type.

As such, we expected that Tier 1 agents would have a higher rate of prior weaponization efforts than non-Tier 1 agents. In our analysis, we searched publicly available literature on state weaponization programs as well as instances conducted by lone actors or groups for corroboration of prior weaponization attempts and successes. It should be noted that public information on weaponization programs is limited, and that our data only reflects weaponization efforts that we were able to reasonably source (Supplementary Table S1). Our review demonstrated that 80.0% of Tier 1 agents have either been weaponized or researched for weaponization, compared to 52.1% of non-Tier 1 agents (Table 4; Figure 5). Statistical analysis demonstrated that Tier 1 agents are 3.68 times more likely to have been the subject of prior weaponization efforts than non-Tier 1 agents (Table 5). We also suspected that plants were skewing this analysis; however, when plants were excluded, Tier 1 agents still have an OR above threshold and are 3.30 times more likely to have been subjects of prior weaponization efforts than non-Tier 1 agents. While prior weaponization was still the narrow majority result for non-Tier 1 agents, we considered the reliability of reports of this information to be low on average, since it is often based on personal accounts and anecdotes. Given the combination of the significant difference between groups and the difficulty of conclusively establishing weaponization efforts, we attribute prior weaponization efforts as a typical characteristic of only Tier 1 BSAT (Figure 6).

### 3.4 The Prototypical Tier 1 and non-Tier 1 BSAT

Collectively, the above analysis allowed us to generate a prototype model of both Tier 1 and non-Tier 1 BSAT, the overall findings of which are detailed in Figure 6. Importantly, the distinguishing features separating the two prototypes are:

• Vaccine development
• Therapeutic development, availability, and efficacy
• Untreated mortality rate
• BSAT type
• Prior weaponization consideration, attempts, and achievement

Of these, BSAT type was the least discerning, with bacteria being more represented in Tier 1 but viruses well represented in both prototypes.

Three facets of these prototypes merit further discussion: vaccines, BSAT type, and host tropism. Our initial vaccine analysis seemed counterintuitive; vaccines are generally indicative of an ability to prevent outbreaks; yet more vaccines are developed and available for Tier 1 BSAT than non-Tier 1 BSAT. Our explanation for this observation is that because Tier 1 BSAT are perceived to pose greater threat to public health, more resources have been invested in developing vaccines for Tier 1 BSAT. While this has led to greater availability of vaccines for Tier 1 agents, this fact has not been compelling enough to consider these agents sufficiently de-risked such that they can be de-tiered. This is possibly an artifact of the recency of Tiering itself (starting in 2012) and the minimal vaccine advancements made for Tier 1 agents since that time, but could also indicate that in the government’s estimation vaccine availability does not reduce risk sufficiently for de-Tiering in these cases. The latter seems most likely, and if so, is undoubtedly correlated with the additional factor of prior weaponization efforts.

To the factor of BSAT type, around 270 viral species (Forni et al., 2022) and 1,500 bacterial species (Bartlett et al., 2022) are known to infect humans. This contrasts with 11,273 species of taxonomically classified viruses (International Committee on Taxonomy of Viruses, 2024) and 35,498 taxonomically classified species of bacteria (Schloss and Handelsman, 2004) (though researchers estimate the true number of species is much higher). When these values are compared to the actual makeup of the SATL, there is a clear skew towards viruses. We hypothesize that this skew may be influenced by human perception of risk; viruses are viewed as having a higher risk of causing a pandemic (Adalja et al., 2019). As the drafting of the SATL is inherently subjective in that it is influenced by SMEs whose opinions shape the final form of the list, we cannot ignore the role that perceived versus actual risk plays in the list. This may represent biases of biological function, transmission dynamics, therapeutic/sanitation technology, human perception, genome sampling and construction capabilities, and more.

In Tier 1, there is a skew toward bacteria as compared to the overall SATL. This overrepresentation seems most likely to be a vestige of early bioweapons research efforts, which found more success in the weaponization of bacteria. As shown above, retention of agents on the SATL is clear for those that have been the focus of prior weaponization efforts. Given this, a variety of factors may have resulted in more “success” of these weaponization efforts for bacteria in comparison to other microorganisms and toxins prior to the enactment of the Biological and Toxins Weapons Convention (BTWC) (Tuzmukhamedov, 1972). These include, but are not limited to, ability to cultivate, availability of antibiotics, availability of vaccines, stability, etc. It may also be possible that the rapid discovery of viruses and the more common emergence of new viruses in the last 30 years has diluted the non-Tier 1 proportion of bacteria, while this effect is muted in Tier 1 because it has not changed much since inception.

Finally, the observed bias toward human-affecting agents and toxins represents anthropocentrism in perceived risk evaluation, as might be expected. But it is worth noting that the effects of destabilized economies and food chains may warrant more serious consideration in perceived risk evaluation. Human illness only represents one form of suffering that is possible due to BSAT events, and we posit that the carnality and relatability of human illness may lead to underestimation of the potential suffering elicited by animal- and plant-affecting BSAT events.

## 4 Framework for de-risking BSAT: priority experiments

We propose an increased effort to perform targeted experiments aimed at providing foundational knowledge of BSAT. In order to balance the threat with the benefits, our proposed solution recommends a set of priority experiments that will aid the government in any future decisions of de-tiering and de-listing agents and toxins. Based on our above analyses, we believe that experiments examining BSAT morbidity, mortality, infectious or toxic dose, and ease of genetic alteration are the most viable. To these ends, studies of basic structural biology, biochemistry, host-pathogen interactions, and immunology will give insight into the above characteristics as well as provide additional foundational understanding of these pathogens and toxins. These experiments will provide key, consistent information about certain characteristics of BSAT that we believe are crucial for de-risking them.

Our prototype analysis revealed characteristics that are seemingly important in FSAP’s evaluation of Tier 1 status, and filling in the gaps for each agent regarding our prototype characteristics would presumably be the first step to de-risking agents. Furthermore, for prototyping to be an effective perspective of what constitutes a typical Tier 1 and non-Tier 1 agent, and to have that standard apply to current and emerging agents, we must ensure that robust and informative data is readily available for all agents, which can then be used to de-risk and potentially de-tier and/or de-list agents. However, we recognize that direct measurement of such criteria is not always feasible given dual-use research of concern (DURC) and BTWC considerations, as well as the requirement for specialized facilities to conduct such experiments.

**FIGURE 7:**
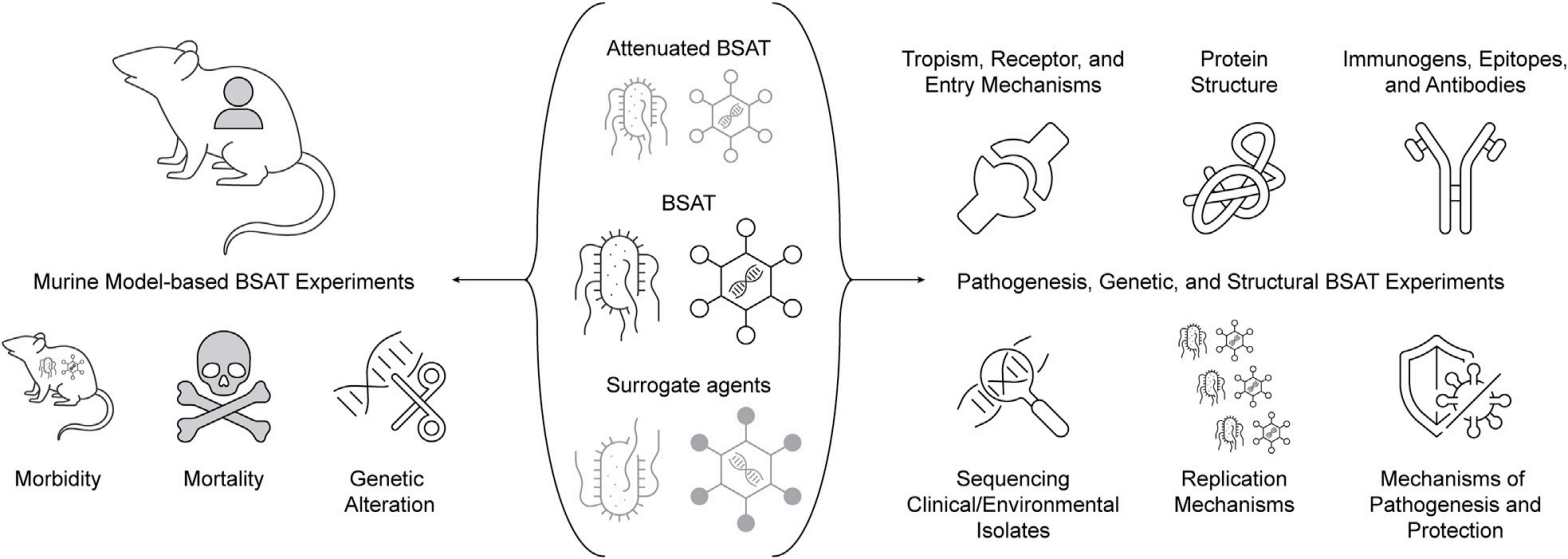
Priority experiments for BSAT risk reduction.

As such, we propose a set of priority experiments that will use either murine models, biochemical pathogenesis, genetic, or structural biology techniques to elucidate and approximate the abovementioned characteristics (Figure 7). These experiments are proposed with the goal of not only informing decisions for the SATL, but also having applicability to pathogen and toxin research more generally. The results of these experiments would clearly be valuable for updating proposed decision framework models for BSAT inclusion/exclusion deliberations (to which we will return later). We recommend that relevant government agencies (such as HHS, DHS, etc.) coordinate with FSAP to seek out registered laboratories and facilitate funding of these experiments to enable risk reduction of BSAT. As with any recommendation, we acknowledge the funding challenge this presents, and propose that a potential solution for FSAP is to seek inroads with existing programs; examples include NIAID’s prototype pathogen model initiative and DHS’ PANTHR program. It is worth noting that these recommendations have similarity (albeit are more targeted) to some of those proposed in 2010 by a committee of the National Research Council, and have remained largely unaddressed (National Academies, 2010).

### 4.1 Murine models for BSAT research

The recent development of humanized murine models allows for testing of human-affecting agents and toxins with more human-relevant results than previously attainable, and is an important avenue for reduction of dual-use concern (Allen et al., 2019). This is true even while acknowledging that these models still have limitations in their prediction of human clinical outcomes (Walsh et al., 2017). Extending this approach to the development of murine models for the study of classes of agents and toxins as a method for studying BSAT would clearly have translational value for the field(s) at large, and we thus view humanized murine models for the study of BSAT as a high-value and low perceived risk approach. Such models would then allow morbidity and mortality evaluation both qualitatively and temporally in ways unattainable through vaccination and challenge trialing in humans. Further, *in vivo* viability studies of genetically altered agents and toxins would be enabled by such models, including mutational strategies, targeted genetic modifications, and chimeric agents and toxins. This strategy does not necessitate using BSAT, but instead could use related surrogate agents or attenuated versions of BSAT. Pairing this approach with meta-analysis studies of the amenability of classes of agents and toxins to genetic alteration through literature review and interviews with experts would provide key information on the ease and feasibility of genetically altering BSAT, informing perceived risk approximation.

One example of the potential benefits of humanized murine models is the success of those developed for influenza A virus (IAV). Researchers aimed to increase the function of the innate and adaptive immune response in modified mice to IAV; to do so, they infected human PBMC-reconstituted immunodeficient mice with H1N1 and H5N1 IAV, and then tracked disease progression and outcomes. These methods then enabled the development of novel therapeutics and vaccines against IAV, which are currently under investigation for their utility. As such, the application of humanized murine models has potential downstream applications for de-risking of both BSAT and non-listed agents and toxins, which can help to justify the cost and potential risk associated with the creation of such models (Krishnakumar et al., 2019).

### 4.2 Pathogenesis, genetic, and structural approaches for BSAT research

We also recommend development and implementation of pan-experiments that are applicable to many agents on the SATL, building off of NIAID’s prototype pathogen model for pandemic preparedness (Deschamps et al., 2023). It is our belief that the examination of the following experiments in all BSAT will prove an appropriate starting point for further evaluation and consideration for de-risking: protein structure elucidation, sequencing of clinical and environmental isolates, determination of primary immunogens, cell tropism and receptor requirements, elucidation of replication mechanisms, toxin binding partners, and development of monoclonal antibodies. All of the aforementioned goals provide valuable insight into the way pathogens harm their hosts and replicate, and as such indirectly equate to the risk presented by each pathogen. Protein structure elucidation could be accomplished via techniques like cryo-electron microscopy, and would allow researchers to gain insight into the enzymatic processes involved in pathogenesis (Cheng, 1979; Thompson and Kovall, 2019). Similarly, determination of a primary immunogen(s) and protective epitopes for each BSAT would allow for a greater understanding of how an infection propagates within its host and gives a starting point for vaccine and therapeutic development. Primary immunogen detection could be elucidated through either immunohistology investigation or solid phase immunoassays (Butler, 2000). Regarding cell tropism, receptor requirements, and replication mechanisms, understanding the proteins (both host and pathogen) that are essential for cellular susceptibility and permissivity would be useful in gaining a more comprehensive view of pathogen threat (Morizono and Chen, 2011). Finally, with knowledge of primary immunogens and receptor binding interactions in mind, the development of sterilizing monoclonal antibodies is a significant step in de-risking an agent or toxin, the production of which could direct therapeutic development (StatPearls, 2023).

NIAID’s prototype pathogen model centers around viruses, but the concept of using a single agent or toxin as a surrogate agent seems transposable beyond this group as well. We recommend that FSAP work with NIAID to determine appropriate surrogates for all Tier 1 agents, and to then encourage, through grant funding, research on sequences of primary clinical isolates, viral entry protein structure/arrangement, host receptors and entry mechanisms, and viral pathogenesis mechanisms (Deschamps et al., 2023). The results of these experiments would serve as a foundation of knowledge that could be used to de-risk BSAT in ways that limit DURC.

## 5 Threshold (goal) development and utilization for de-risking BSAT

By pairing the above criteria and factors of the prototype BSAT model with the de-risking priority experiments, we can begin to formalize approaches to inclusion/exclusion decisions for the SATL. The government’s recently developed data sheets and decision framework are a natural fit for such integration (Pillai et al., 2023; Pillai et al., 2022a; Pillai et al., 2022b). We propose using the data sheets as not only an expansive resource for overall evaluation of BSAT perceived risk, but also in identifying key criteria and factors that lack recent or reliable data, are amenable to de-risking, and are most influential in decisions for inclusion/exclusion from the SATL. The latter has already been considered (Pillai et al., 2022a), and is reflected in our selection of criteria and factors in the prototype BSAT model. By developing a risk reduction “guide” for BSAT, the data sheets could be a key tool for threat reduction.

We envision a de-risking overlay on the existing data sheets, an example of which we draft in Table 6. This approach is a composite of the various criteria identified throughout this manuscript that we believe are most formative for deliberations on inclusion/exclusion of BSAT. Key to the approach is the delineation between factual (but currently poorly defined) information and directly reducible perceived risks. For example, the infectious dose required to infect 50% of healthy adult humans (ID_50_) of an organism is some fixed value, but for most Tier 1 BSAT is approximated by subject matter experts, since the human experimental data to determine this value is not available (for good reason). As such, information that increases the certainty of the true ID_50_ and that indicates the value to be higher than previously expected (so, more dose required) is an indirect de-risking finding even though the factual information has not changed. As another example, countermeasures are a category with clearly manipulable perceived risk level; the development of a more effective vaccine or drug than previously available is a clear de-risking event.

**TABLE 6:** Overlaying risk reduction thresholds on FSAP BSAT data sheets.

Score	Definition	Threshold
Ability to Genetically Manipulate or Alter The degree of difficulty of the techniques required to create a more virulent, transmissible, environmentally stable or countermeasure-resistant strain		
0	No known method	
2	Very difficult (e.g., negative strand RNA viruses)	De-Tier/de-list
4	Highly difficult (e.g., positive strand RNA viruses, gene reassortment or reverse genetics available)	Threshold
6	Moderately difficult (e.g., DNA viruses and intracellular bacteria)	
8	Low difficulty (e.g., plasmid insertion for bacteria)	No change
10	No directed genetic manipulation required	
Availability of Medical Countermeasures The availability of efficacious medical treatments/countermeasures and extent to which they can be rapidly deployed in response to a public health emergency		
0	No MCMs required or would be deployed	
2	Widely available and easy to deploy efficiently	De-Tier/de-list
4	Widely available but difficult to deploy efficiently or lacks efficacy	Threshold
6	Available in limited quantities	
8	Experimental treatment in development	No change
10	No treatment beyond supportive care available	
Case Fatality Rate The number of deaths from the disease per 100 diagnosed cases (or number of known cases)		
0	Close to 0%	De-Tier/de-list
2	1%–9%; or <10 known cases	Threshold
4	10%–29%	
6	30%–39%	
8	40%–49%	No change
10	50%–100%	
Infectious dose (ID50) The dose or amount of agent (in cfu or pfu as appropriate) required to infect 50% of a healthy adult human population by inhalation or ingestion		
0	Not infectious by inhalation or ingestion	
2	>10,000	De-Tier/de-list
4	1,000–10,000	Threshold
6	100–1,000	
8	10–100	No change
10	1–10	

Adapted from https://www.frontiersin.org/articles/10.3389/fbioe.2022.756586/full

For Tier 1 BSAT, thresholds on the scales of each criterion can be determined where the perceived risk would be considered acceptable for de-tiering and/or de-listing. While the thresholds for each organism may differ (because of other criteria on the data sheets), we expect that this exercise is relatively easily accomplished. Thresholds could be fixed (ex: in the lower half of a criteria’s scale, as in Table 6) or movement-based (ex: reducing a criterion by two levels from the current ranking of the data sheet). These thresholds, then, can also serve as goals, to which de-risking priority experiments can be tuned. Whether all or a proportion of the thresholds must be accomplished in order to de-tier or de-list a BSAT is a topic for discussion by ISATTAC and FSAP, but we would recommend starting by choosing reasonably attainable thresholds. Then, if at least one threshold is attained, to consider de-tiering that agent or toxin; and if at least three are attained, to consider de-listing that agent or toxin altogether. It is worth noting that the government’s proposed approach relies more heavily on statistical thresholds (Pillai et al., 2023; Pillai et al., 2022a; Pillai et al., 2022b). Our approach is not meant to supplant that, but rather, to extract the most meaningful criteria, provide simple metrics to enable more targeted de-risking, and to augment efforts to determine whether a BSAT should be tiered or listed (or not).

Providing known off-ramps for agents and toxins would ultimately make the work of FSAP easier by reducing the vagueness of these decisions, making it easier for the public to understand and contribute to dialog about BSAT decisions, and hopefully shrinking the SATL in the long-term (which would represent an incredible accomplishment in risk reduction). These approaches are potential solutions to bridging list- and risk-based biosecurity, providing a middle ground between current list-based infrastructure and the biosecurity community’s growing interest in risk-based and pathogen agnostic approaches (Subcommittee on Biological Defense Research and Development Committee on Homeland and National Security of the National Science and Technology Council, 2017; Leiser et al., 2021; Lim and Popescu, 2024). Adoption of such approaches would greatly improve the efficiency of the SARs, through both reducing the volume of regulatory oversight efforts and enabling de-risking scientific research.

## 6 Conclusion

In this manuscript, we have synthesized multiple approaches to build a pathway for de-risking the most dangerous biological agents and toxins. By combining a prototype pathogen approach, priority experiments, and selection thresholds, we provide a mechanism for enabling targeted risk reduction strategies. Notably, adoption of these goals would have benefits expanding well beyond the select agent regulations, and could be formative for pathogen research, threat modeling, and biosecurity practice more broadly. Overall, we believe that the approaches presented here represent a significant opportunity that is long overdue; to transition the SATL from its current mold of concern and stasis to a generative tool for action with the goal of actively reducing the biosecurity risks posed by select agents and toxins.

## Data Availability

The original contributions presented in the study are included in the article/Supplementary Material, further inquiries can be directed to the corresponding author.
